# The "Majority Illusion" in Social Networks

**DOI:** 10.1371/journal.pone.0147617

**Published:** 2016-02-17

**Authors:** Kristina Lerman, Xiaoran Yan, Xin-Zeng Wu

**Affiliations:** 1 USC Information Sciences Institute, Marina del Rey, CA, United States of America; 2 Department of Physics and Astronomy, University of Southern California, Los Angeles, CA, United States of America; Université Toulouse 1 Capitole, FRANCE

## Abstract

Individual’s decisions, from what product to buy to whether to engage in risky behavior, often depend on the choices, behaviors, or states of other people. People, however, rarely have global knowledge of the states of others, but must estimate them from the local observations of their social contacts. Network structure can significantly distort individual’s local observations. Under some conditions, a state that is globally rare in a network may be dramatically over-represented in the local neighborhoods of many individuals. This effect, which we call the “majority illusion,” leads individuals to systematically overestimate the prevalence of that state, which may accelerate the spread of social contagions. We develop a statistical model that quantifies this effect and validate it with measurements in synthetic and real-world networks. We show that the illusion is exacerbated in networks with a heterogeneous degree distribution and disassortative structure.

## Introduction

An individual’s attitudes and behaviors are shaped by his or her perceptions of the choices, attitudes, and behaviors of others [1–6]. This phenomenon is manifested daily in the decisions people make to adopt a new technology [7, 8] or idea [5, 9], listen to music [3], engage in risky behavior [10], abuse alcohol [11, 12], or join a social movement [1, 2]. As a result, a variety of behaviors are said to be “contagious”, because they spread through the population as people perceive others adopting the behavior and then adopt it themselves. In some cases, “social contagion” will spread from a small number of initial adopters to a large portion of the population, resulting in a fad, hit song, successful political campaign, or a prevailing social norm. Researchers have linked the onset of such global outbreaks to the topology of the underlying network [6, 13], the presence of highly connected individuals [14, 15] and small clusters of inter-connected people [4, 5].

Network structure, however, can systematically bias social perceptions and the inferences people make about their peers. Socially connected individuals tend to be similar [16]. This exposes people to a biased sample of the population, giving rise to the “selective exposure” [17] effect that leads individuals to overestimate the prevalence of their features in a population [18]. Moreover, individuals may selectively divulge or conceal their attributes or behaviors from peers, especially if these deviate from prevailing norms. Such “selective disclosure” [17, 19] will further bias social perceptions, leading individuals to incorrectly infer the prevalence of the behavior in the population. Social perception biases can alter the dynamics of social contagions and stabilize unpopular attitudes and behaviors [20, 21].

Beyond the effects described above, network structure may further distort social perceptions by biasing individual’s observations. One of these network biases is the friendship paradox, which states that, on average, most people have fewer friends than their friends have [22]. Despite its almost nonsensical nature, the friendship paradox has been used to design efficient strategies for vaccination [23], social intervention [24], and early detection of contagious outbreaks [25, 26]. In a nutshell, rather than monitoring random people to catch a contagious outbreak in its early stages, the friendship paradox suggests monitoring their random network neighbors, because they are more likely to be better connected and not only to get sick earlier, but also to infect more people once sick. Recently, friendship paradox was generalized for attributes other than degree, i.e., number of network neighbors. For example, your co-authors are cited more often than you [27], and the people you follow on Twitter post more frequently than you do [28]. In fact, any attribute that is correlated with degree will produce a paradox [27, 29].

We describe a novel variation of the friendship paradox that is essential for understanding social contagion. The paradox applies to networks in which individuals have attributes, in the simplest case a binary attribute, such as “has red hair” vs “does not have red hair,” “purchased an iPhone” vs “did not purchase an iPhone,” “Democrat” vs “Republican.” We refer to individuals with this attribute as “active”, and the rest as “inactive.” We show that under some conditions many individuals will observe a majority of their neighbors in the active state, even when it is globally rare. For example, though few people have red hair, many may observe that a majority of their friends are red-headed. For this reason, we call this effect the “majority illusion.”

**Fig 1:**
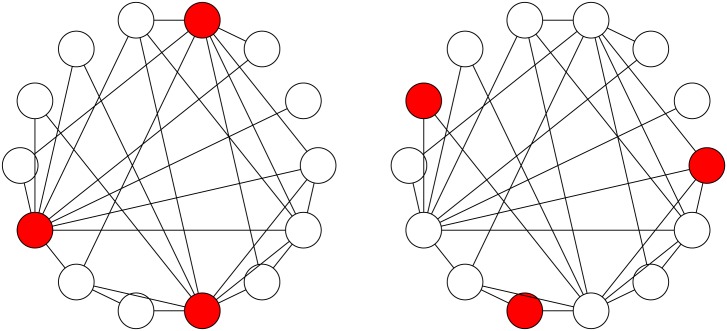
An illustration of the “majority illusion” paradox. The two networks are identical, except for which three nodes are colored. These are the “active” nodes and the rest are “inactive.” In the network on the left, all “inactive” nodes observe that at least half of their neighbors are “active,” while in the network on the right, no “inactive” node makes this observation.

As a simple illustration of the “majority illusion” paradox, consider the two networks in Fig 1. The networks are identical, except for which of the few nodes are colored. Imagine that colored nodes are active and the rest of the nodes are inactive. Despite this apparently small difference, the two networks are profoundly different: in the first network, every inactive node will examine its neighbors to observe that “at least half of my neighbors are active,” while in the second network no node will make this observation. Thus, even though only three of the 14 nodes are active, it appears to all the inactive nodes in the first network that most of their neighbors are active.

The “majority illusion” can dramatically impact collective phenomena in networks, including social contagions. One of the more popular models describing the spread of social contagions is the threshold model [2, 13, 30]. At each time step in this model, an inactive individual observes the current states of its *k* neighbors, and becomes active if more than *ϕk* of the neighbors are active; otherwise, it remains inactive. The fraction 0 ≤ *ϕ* ≤ 1 is the activation *threshold*. It represents the amount of social proof an individual requires before switching to the active state [2]. Threshold of *ϕ* = 0.5 means that to become active, an individual has to have a majority of neighbors in the active state. Though the two networks in Fig 1 have the same topology, when the threshold is *ϕ* = 0.5, all nodes will eventually become active in the network on the left, but not in the network on the right. This is because the “majority illusion” alters local neighborhoods of the nodes, distorting their observations of the prevalence of the active state. Thus, “majority illusion” provides an alternate mechanism for social perception biases. For example, if heavy drinkers also happen to be more popular (they are the red nodes in the figure above), then, while most people drink little at parties, many people will examine their friends’ alcohol use to observe a majority drinking heavily. This may explain why adolescents overestimate their peers’ alcohol consumption and drug use [11, 12, 31].

The magnitude of the “majority illusion” paradox, which we define as the fraction of nodes more than half of whose neighbors are active, depends on structural properties of the network and the distribution of active nodes. Network configurations that exacerbate the paradox include those in which low-degree nodes tend to connect to high-degree nodes (i.e., networks are disassortative by degree). Activating the high-degree nodes in such networks biases the local observations of many nodes, which in turn impacts collective phenomena emerging in networks, including social contagions and social perceptions. We develop a statistical model that quantifies the strength of this effect in any network and evaluate the model using synthetic networks. These networks allow us to systematically investigate how network structure and the distribution of active nodes affect observations of individual nodes. We also show that structure of many real-world networks creates conditions for the “majority illusion” paradox.

## Materials and Methods

We used the configuration model [32, 33], as implemented by the SNAP library (https://snap.stanford.edu/data/) to create a scale-free network with a specified degree sequence. We generated a degree sequence from a power law of the form *p*(*k*)∼*k*^−*α*^. Here, *p*_*k*_ is the fraction of nodes that have *k* half-edges. The configuration model proceeded by linking a pair of randomly chosen half-edges to form an edge. The linking procedure was repeated until all half-edges have been used up or there were no more ways to form an edge.

To create Erdős-Rényi-type networks, we started with *N* = 10,000 nodes and linked pairs at random with some fixed probability. These probabilities were chosen to produce average degree similar to the average degree of the scale-free networks.

The statistics of real-world networks we studied, including the collaboration network of high energy physicist (HepTh), Human protein–protein interactions network from Reactome project (http://www.reactome.org/pages/download-data/), Digg follower graph (DOI:10.6084/m9.figshare.2062467), Enron email network (http://www.cs.cmu.edu/∼enron/), Twitter user voting graph [34], and a network of political blogs (http://www-personal.umich.edu/∼mejn/netdata/) are summarized in Table 1.

**Table 1:** Network properties. Size of networks studied in this paper, along with their average degree 〈*k*〉 and degree assortativity coefficient *r*_*kk*_.

*network*	*nodes*	*edges*	〈*k*〉	*r*_*kk*_
HepTh	9,877	25,998	5.26	0.2679
Reactome	6,327	147,547	46.64	0.2491
Digg	27,567	175,892	12.76	0.1660
Enron	36,692	367,662	20.04	−0.1108
Twitter	23,025	336,262	29.21	−0.1375
Political blogs	1,490	19,090	25.62	−0.2212

## Results

A network’s structure is partly specified by its degree distribution *p*(*k*), which gives the probability that a randomly chosen node in an undirected network has *k* neighbors (i.e., degree *k*). This quantity also affects the probability that a randomly chosen edge is connected to a node of degree *k*, otherwise known as neighbor degree distribution *q*(*k*). Since high-degree nodes have more edges, they will be over-represented in the neighbor degree distribution by a factor proportional to their degree; hence, *q*(*k*) = *kp*(*k*)/〈*k*〉, where 〈*k*〉 is the average node degree.

Networks often have structure beyond that specified by their degree distribution: for example, nodes may preferentially link to others with a similar (or very different) degree. Such degree correlation is captured by the joint degree distribution *e*(*k*, *k*′), the probability to find nodes of degrees *k* and *k*′ at either end of a randomly chosen edge in an undirected network [35]. This quantity obeys normalization conditions ∑_*kk*′_ *e*(*k*, *k*′) = 1 and ∑_*k*′_ *e*(*k*, *k*′) = *q*(*k*). Globally, degree correlation in an undirected network is quantified by the assortativity coefficient, which is simply the Pearson correlation between degrees of connected nodes:

$$\begin{array}{c} r{}_{kk}=\frac{1}{\sigma_{q}^{2}}\sum_{k,k^{\prime }}kk^{\prime }[e\left(k,k^{\prime }\right)-q\left(k\right)q\left(k^{\prime }\right)]=\frac{1}{\sigma_{q}^{2}}[(\sum_{k,k^{\prime }}kk^{\prime }e\left(k,k^{\prime }\right))-{\left\langle k\right\rangle }_{q}^{2}]. \end{array}$$ (1)

Here, $\sigma_{q}^{2}=\sum_{k}k^{2}q\left(k\right)-{\left[\sum_{k}kq(k)\right]}^{2}$. In assortative networks (*r*_*kk*_ > 0), nodes have a tendency link to similar nodes, e.g., high-degree nodes to other high-degree nodes. In disassortative networks (*r*_*kk*_ < 0), on the other hand, they prefer to link to dissimilar nodes. A star composed of a central hub and nodes linked only to the hub is an example of a disassortative network.

We can use Newman’s edge rewiring procedure [35] to change a network’s degree assortativity without changing its degree distribution *p*(*k*). The rewiring procedure randomly chooses two pairs of connected nodes and swaps their edges if doing so changes their degree correlation. This can be repeated until desired degree assortativity is achieved.

The configuration of attributes in a network is specified by the joint probability distribution *P*(*x*, *k*), the probability that node of degree *k* has an attribute *x*. In this work, we consider binary attributes only, and refer to nodes with *x* = 1 as active and those with *x* = 0 as inactive. The joint distribution can be used to compute *ρ*_*kx*_, the correlation between node degrees and attributes:

$$\begin{array}{ccc} \rho_{kx} & \equiv & \frac{1}{\sigma_{x}\sigma_{k}}\sum_{x,k}xk[P(x,k)-P(x)p(k)]\\ & = & \frac{1}{\sigma_{x}\sigma_{k}}\sum_{k}k[P(x=1,k)-P(x=1)p(k)]=\frac{P(x=1)}{\sigma_{x}\sigma_{k}}[{\left\langle k\right\rangle }_{x=1}-\langle k\rangle ]. \end{array}$$ (2)

In the equations above, *σ*_*k*_ and *σ*_*x*_ are the standard deviations of the degree and attribute distributions respectively, and 〈*k*〉_*x* = 1_ is the average degree of active nodes.

Randomly activating nodes creates a configuration with *ρ*_*kx*_ close to zero. We can change it by swapping attribute values among the nodes. For example, to increase *ρ*_*kx*_, we randomly choose nodes *v*_1_ with *x* = 1 and *v*_0_ with *x* = 0 and swap their attributes if the degree of *v*_0_ is greater than the degree of *v*_1_. We can continue swapping attributes until desired *ρ*_*kx*_ is achieved (or it no longer changes).

### “Majority Illusion” in Synthetic and Real-world Networks

Synthetic networks allow us to systematically study how network structure affects the strength of the “majority illusion” paradox. First, we looked at networks with a highly heterogeneous degree distribution, which contain a few high-degree hubs and many low-degree nodes. Such networks are usually modeled with a scale-free degree distribution of the form *p*(*k*)∼*k*^−*α*^. To create a heterogeneous network, we first sampled a degree sequence from a distribution with exponent *α*, where exponent *α* took three different values (2.1, 2.4, and 3.1), and then used the configuration model to create an undirected network with *N* = 10,000 nodes and that degree sequence. We used the edge rewiring procedure described above to create a series of networks that have the same degree distribution *p*(*k*) but different values degree assortativity *r*_*kk*_. Then, we activated a fraction *P*(*x* = 1) = 0.05 of nodes and used the attribute swapping procedure to achieve different values of degree–attribute correlation *ρ*_*kx*_.

**Fig 2:**
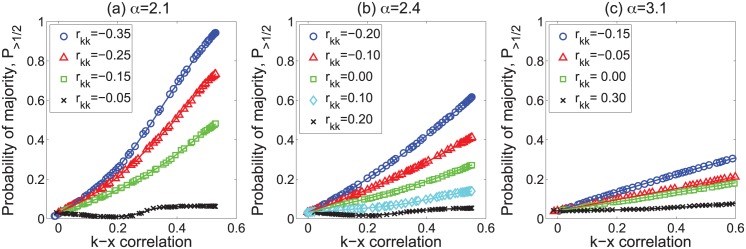
“Majority illusion” in scale-free networks. Plots show the magnitude of the illusion in scale-free networks as a function of degree–attribute correlation *ρ*_*kx*_ and for different values of degree assortativity *r*_*kk*_. Each network has 10,000 nodes and degree distribution of the form *p*(*k*)∼*k*^−*α*^. The fraction of active nodes in all cases is 5%. The lines represent calculations using the statistical model of Eq (5).

Fig 2 shows the fraction of nodes with more than half of active neighbors in these scale-free networks as a function of the degree–attribute correlation *ρ*_*kx*_. The fraction of nodes experiencing the “majority illusion” can be quite large. For *α* = 2.1, 60%–80% of the nodes will observe that more than half of their neighbors are active, even though only 5% of the nodes are, in fact, active. The “majority illusion” is exacerbated by three factors: it becomes stronger as the degree–attribute correlation increases, and as the network becomes more disassortative (i.e., *r*_*kk*_ decreases) and heavier-tailed (i.e., *α* becomes smaller). However, even when *α* = 3.1, under some conditions a substantial fraction of nodes will experience the paradox. The lines in the figure show show theoretical estimates of the paradox using Eq (5), as described in the next subsection.

**Fig 3:**
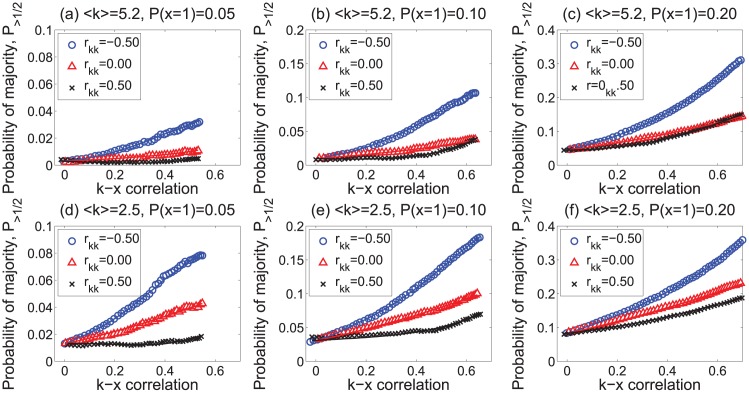
“Majority illusion” in random networks. Magnitude of the illusion in Erdős-Rényi-type random networks as a function of degree–attribute correlation *ρ*_*kx*_ and for different values of degree assortativity *r*_*kk*_. Each network has 10,000 nodes with 〈*k*〉 = 5.2 (top row) or 〈*k*〉 = 2.5 (bottom row), and different fractions of active nodes. The lines represent calculations using the statistical model of Eq (5).

“Majority illusion” can also be observed in networks with a more homogeneous, e.g., Poisson, degree distribution. We used the Erdős-Rényi model to generate networks with *N* = 10,000 and average degrees 〈*k*〉 = 5.2 and 〈*k*〉 = 2.5. We randomly activated 5%, 10%, and 20% of the nodes, and used edge rewiring and attribute swapping to change *r*_*kk*_ and *ρ*_*kx*_ in these networks. Fig 3 shows the fraction of nodes in the paradox regime. Though much reduced compared to scale-free networks, we still observe some amount of the paradox, especially in networks with a greater fraction of active nodes.

We also examined whether “majority illusion” can be manifested in real-world networks. We looked at six different networks: the co-authorship network of high energy physicists (HepTh) [36], protein-protein interactions network (Reactome) [37], social media follower graphs (Digg [38] and Twitter [34]), Enron email network [39], and the network representing links between political blogs (blogs) [40]. All six networks are undirected. To make the Digg and Twitter follower graphs undirected, we kept only the mutual follow links, and further reduced the graph by extracting the largest connected component. For Enron email network, we removed duplicate email communication links between users. The degree assortativity of these networks spans a broad range, from *r*_*kk*_ = 0.27 (HepTh) to *r*_*kk*_ = −0.22 (political blogs).

**Fig 4:**
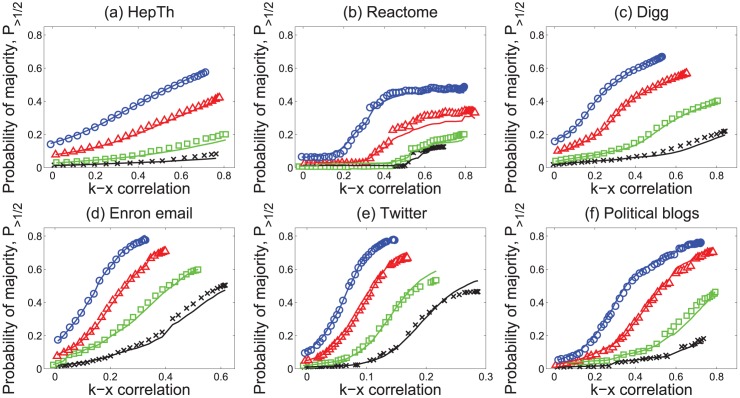
“Majority illusion” in real-world networks. Magnitude of the illusion in real-world networks as a function of degree–attribute correlation *ρ*_*kx*_ for different fraction of active nodes *P*(*x* = 1). The lines represent calculations using the statistical model of Eq (5). The plots are arranged in order of degree assortativity, from highest (a) to lowest (f). Blue circles correspond to the fraction of active nodes *P*(*x* = 1) = 0.3, red triangles to *P*(*x* = 1) = 0.2, green squares to *P*(*x* = 1) = 0.1, and black crosses to *P*(*x* = 1) = 0.05.

Fig 4 shows the fraction of nodes experiencing the “majority illusion” for different fractions of active nodes *P*(*x* = 1) = 0.05, 0.1, 0.2 and 0.3. As degree–attribute correlation *ρ*_*kx*_ increases (using the attribute swapping procedure), a substantial fraction of nodes experience the paradox in almost all networks. The effect is larger in disassortative political blogs, Twitter and Enron email networks, where for high enough correlation, as many as 60%–70% of nodes have more than half of their neighbors in the active state, even though only 20% of the nodes are active. The effect also exists in the Digg network of mutual followers, and to a lesser degree in the HepTh co-authorship and Reactome protein interactions network. Although positive degree assortativity reduces the magnitude of the effect, compared with synthetic networks, local perceptions of nodes in real-world networks can also be substantially skewed. If the attribute represents an opinion, under some conditions, even a minority opinion can appear to be extremely popular locally.

### Quantifying the “Majority Illusion” in Networks

Having demonstrated empirically some of the relationships between “majority illusion” and network structure, we next develop a model that includes network properties in the calculation of paradox strength. Like the friendship paradox, the “majority illusion” is rooted in differences between degrees of nodes and their neighbors [22, 41]. These differences result in nodes observing that, not only are their neighbors better connected [22] on average, but that they also have more of some attribute than they themselves have [28]. The latter paradox, which is referred to as the generalized friendship paradox, is enhanced by correlations between node degrees and attribute values *ρ*_*kx*_ [27]. In binary attribute networks, where nodes can be either active or inactive, a configuration in which higher degree nodes tend to be active causes the remaining nodes to observe that their neighbors are more active than they are (S1 File).

While heterogeneous degree distribution and degree–attribute correlations give rise to friendship paradoxes even in random networks, other elements of network structure, such as degree assortativity *r*_*kk*_ [42], may also affect observations nodes make of their neighbors. To understand why, we need a more detailed model of network structure that includes correlation between degrees of connected nodes *e*(*k*, *k*′). Consider a node with degree *k* that has a neighbor with degree *k*′ and attribute *x*′. The probability that the neighbor is active is:

$$\begin{array}{c} P\left(x^{\prime }=1|k\right)=\sum_{k^{\prime }}P\left(x^{\prime }=1|k^{\prime }\right)P\left(k^{\prime }|k\right)=\sum_{k^{\prime }}P\left(x^{\prime }=1|k^{\prime }\right)\frac{e(k,k^{\prime })}{q(k)}. \end{array}$$ (3)

In the equation above, *e*(*k*, *k*′) is the joint degree distribution. Globally, the probability that any node has an active neighbor is

$$\begin{array}{ccc} P(x^{\prime }=1) & = & \sum_{k}P\left(x^{\prime }=1|k\right)p\left(k\right)=\sum_{k}\sum_{k^{\prime }}P\left(x^{\prime }=1|k^{\prime }\right)\frac{e(k,k^{\prime })}{q(k)}p\left(k\right)\\ & = & \sum_{k}\sum_{k^{\prime }}\frac{P(x^{\prime }=1,k^{\prime })}{p(k^{\prime })}e\left(k,k^{\prime }\right)\frac{\left\langle k\right\rangle }{k}=\sum_{k^{\prime }}\frac{P(x^{\prime }=1,k^{\prime })}{q(k^{\prime })}\sum_{k}\frac{k^{\prime }}{k}e\left(k,k^{\prime }\right). \end{array}$$

Given two networks with the same degree distribution *p*(*k*), their neighbor degree distribution *q*(*k*) will be the same even when they have different degree correlations *e*(*k*, *k*′). For the same configuration of active nodes, the probability that a node in each network observes an active neighbor *P*(*x*′ = 1) is a function of ∑_*k*,*k*′_(*k*′/*k*)*e*(*k*, *k*′). Since degree assortativity *r*_*kk*_ is a function of ∑_*k*,*k*′_ *kk*′*e*(*k*, *k*′), the two expressions weigh the *e*(*k*, *k*′) term in opposite ways. This suggests that the probability of having an active neighbor increases as degree assortativity decreases and vice versa. Thus, we expect stronger paradoxes in disassortative networks.

To quantify the “majority illusion” paradox, we calculate the probability that a node of degree *k* has more than a fraction *ϕ* of active neighbors, i.e., neighbors with attribute value *x*′ = 1:

$$\begin{array}{c} P_{>\phi }\left(k\right)=\sum_{n>\phi k}^{k}(\begin{array}{c} k\\ n \end{array})P{\left(x^{\prime }=1|k\right)}^{n}{\left[1-P\left(x^{\prime }=1|k\right)\right]}^{k-n}\;. \end{array}$$ (4)

Here *P*(*x*′ = 1|*k*) is the conditional probability of having an active neighbor, given a node with degree *k*, and is specified by Eq (3). Although the threshold *ϕ* in Eq (4) could be any fraction, in this paper we focus on $\phi =\frac{1}{2}$, which represents a straight majority. Thus, the fraction of all nodes most of whose neighbors are active is

$$\begin{array}{c} P_{>\frac{1}{2}}=\sum_{k}p\left(k\right)\sum_{n>\frac{k}{2}}^{k}(\begin{array}{c} k\\ n \end{array})P{\left(x^{\prime }=1|k\right)}^{n}{\left[1-P\left(x^{\prime }=1|k\right)\right]}^{k-n}\;. \end{array}$$ (5)

Using Eq (5), we can calculate the strength of the “majority illusion” paradox for any network whose degree sequence, joint degree distribution *e*(*k*, *k*′), and conditional attribute distribution *P*(*x*|*k*) are known. The solid lines in Figs 2–4 report these calculations for each network. The conditional probability *P*(*x* = 1|*k*) = *P*(*x*′ = 1|*k*′) required to calculate the strength of the “majority illusion” using Eq (5) can be specified analytically only for networks with “well-behaved” degree distributions, such as scale–free distributions of the form *p*(*k*)∼*k*^−*α*^ with *α* > 3 or the Poisson distributions of the Erdős-Rényi random graphs in near-zero degree assortativity. For other networks, including the real world networks with a more heterogeneous degree distribution, we use the empirically determined joint probability distribution *P*(*x*, *k*) to calculate both *P*(*x* = 1|*k*) and *ρ*_*kx*_. For the Poisson-like degree distributions, the probability *P*(*x*′ = 1|*k*′) can be determined by approximating the joint distribution *P*(*x*′, *k*′) as a multivariate normal distribution:

$$\begin{array}{c} \langle P(x^{\prime }|k^{\prime })\rangle =\langle P(x^{\prime })\rangle +\rho_{kx}\frac{\sigma_{x}}{\sigma_{k}}\left(k^{\prime }-\langle k\rangle \right)\;, \end{array}$$

resulting in

$$\begin{array}{c} P\left(x^{\prime }=1|k^{\prime }\right)=\langle x\rangle +\rho_{kx}\frac{\sigma_{x}}{\sigma_{k}}\left(k^{\prime }-\langle k\rangle \right). \end{array}$$

**Fig 5:**
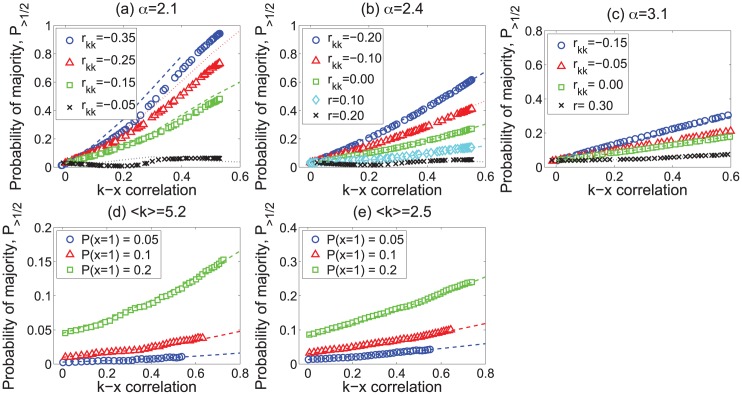
Gaussian approximation. Symbols show the empirically determined fraction of nodes in the paradox regime (same as in Figs 2 and 3), while dashed lines show theoretical estimates using the Gaussian approximation.

Fig 5 reports the “majority illusion” in the same synthetic scale–free networks as Fig 2, but with theoretical lines (dashed lines) calculated using the Gaussian approximation for estimating *P*(*x*′ = 1|*k*′). The Gaussian approximation fits results quite well for the network with degree distribution exponent *α* = 3.1. However, theoretical estimate deviates significantly from data in a network with a heavier–tailed degree distribution with exponent *α* = 2.1. The approximation also deviates from the actual values when the network is strongly assortative or disassortative by degree.

Overall, our statistical model that uses empirically determined joint distribution *P*(*x*, *k*) does a good job explaining most observations. However, the global degree assortativity *r*_*kk*_ is an important contributor to the “majority illusion,” a more detailed view of the structure using joint degree distribution *e*(*k*, *k*′) is necessary to accurately estimate the magnitude of the paradox. As demonstrated in S1 Fig, two networks with the same *p*(*k*) and *r*_*kk*_ (but degree correlation matrices *e*(*k*, *k*′)) can display different amounts of the paradox.

## Conclusion

Local prevalence of some attribute among a node’s network neighbors can be very different from its global prevalence, creating an illusion that the attribute is far more common than it actually is. In a social network, this illusion may cause people to reach wrong conclusions about how common a behavior is, leading them to accept as a norm a behavior that is globally rare. In addition, it may also explain how global outbreaks can be triggered by very few initial adopters. This may also explain why the observations and inferences individuals make of their peers are often incorrect. Psychologists have, in fact, documented a number of systematic biases in social perceptions [43]. The “false consensus” effect arises when individuals overestimate the prevalence of their own features in the population [18], believing their type to be more common. Thus, Democrats believe that most people are also Democrats, while Republicans think that the majority are Republican. “Pluralistic ignorance” is another social perception bias. This effect arises in situations when individuals incorrectly believe that a majority has an attribute or accepts a norm that they themselves do not share. Pluralistic ignorance was invoked to explain why bystanders fail to act in emergencies [44], and why college students tend to overestimate alcohol use among their peers [11, 12, 31].

Psychologists proposed several explanations for these biases (see [17] for a concise review), many based on emotional or cognitive mechanisms. For example, when making social inferences, individuals may use themselves as examples for estimating the states of others (using the “availability” heuristic [45]). This leads them to mistakenly believe that majority shares their attitudes and behaviors. However, if instead of using themselves, individuals use their peers as examples to generalize about the population as a whole, network-based explanations for social perception bias are also possible. “Selective exposure” [17] is one such explanation. Social networks are homophilous [16], meaning that socially linked individuals tend to be similar. Homophily exposes people to a biased sample of the population, creating the false consensus effect [18]. A related mechanism is “selective disclosure” [17, 19], in which people selectively divulge or conceal their attributes or behaviors to peers, especially if these deviate from prevailing norms. This too can bias social perceptions, leading individuals to incorrectly infer the prevalence of the behavior in the population.

The paradox described in this paper provides an alternate network-based mechanism for biases in social perceptions. We showed that under some conditions, individuals will grossly overestimate the prevalence of some attribute, making it appear more popular than it is. We quantified this paradox, which we call the “majority illusion”, and studied its dependence on network structure and attribute configuration. As in the friendship paradox [22, 27–29], “majority illusion” can ultimately be traced to the power of high degree nodes to skew the observations of many others. This is because such nodes are overrepresented in the local neighborhoods of other nodes. This, by itself is not surprising, given than high degree nodes are expected to have more influence and are often targeted by influence maximization algorithms [14]. However, the ability of high degree nodes to bias the observations of others depends on other aspects of network structure. Specifically, we showed that the paradox is much stronger in disassortative networks, where high degree nodes tend to link to low degree nodes. In other words, given the same degree distribution, the high degree nodes in a disassortative network will have greater power to skew the observations of others than those in an assortative network. This suggests that some network structures are more susceptible than others to influence manipulation and the spread of external shocks [13]. Furthermore, small changes in network topology, degree assortativity and degree–attribute correlation may further exacerbate the paradox even when there are no actual changes in the distribution of the attribute. This may explain the apparently sudden shifts in public attitudes witnessed during the Arab Spring and on the question of gay marriage.

The “majority illusion” is an example of class size bias effect. When sampling data to estimate average class or event size, more popular classes and events will be over-represented in the sample, biasing estimates of their average size [46]. Thus, the average class size that students experience at college is larger than the college’s average class size. Similarly, people experience highways, restaurants, and events to be more crowded than they normally are. In networks, sampling bias affects estimates of network structure, including its degree distribution [41, 47]. Our work suggests that network bias also affects an individual’s local perceptions. Further work is required to understand how this bias impacts the dynamics of collective social phenomena.

## Supporting Information

S1 File Friendship paradox. Derivation of the generalized friendship paradox for binary attribute networks.
(PDF)

S1 Fig Structural differences. Strength of the majority illusion in synthetic networks with identical degree sequence and assortativity, but with higher-order structural differences. To create these higher-order structural differences, we used the edge swapping procedure to change the network’s degree correlation matrix *e*(*k*, *k*′).
(EPS)
