# Supplementary material for: The "Majority Illusion" in Social Networks
Source: PLoS One. 2016 Feb 17;11(2):e0147617. doi: 10.1371/journal.pone.0147617 (PMC4757419; doi:10.1371/journal.pone.0147617)
Supplement: S1 File — Derivation of the generalized friendship paradox for binary attribute networks. (PDF) [file pone.0147617.s001.pdf]

S1 Friendship Paradox

The friendship paradox says that the average degree of neighbors of a node of degree k is larger than k [1]. We demonstrate that the paradox holds globally by showing that average neighbor degree $\langle k\rangle_q$ is larger than average node degree $\langle k\rangle$:

$$\langle k\rangle_q - \langle k\rangle = \sum_k \frac{k^2 p(k)}{\langle k\rangle} - \langle k\rangle = \frac{\langle k^2\rangle - \langle k\rangle^2}{\langle k\rangle} = \frac{\sigma_k^2}{\langle k\rangle},$$

Here σ_k is the standard deviation of the degree distribution $p(k)$. Since $\sigma_k \geq 0$, $\langle k\rangle_q - \langle k\rangle \geq 0$. This confirms that the friendship paradox has its origins in the heterogeneous degree distribution, and is more pronounced in networks with larger degree heterogeneity σ_k.

Heterogeneous degree distribution also contributes to nodes perceiving that their neighbors have more of some attribute than they themselves have — what is referred to as the generalized friendship paradox [2]. Let's consider again a network where nodes have a binary attribute x. For convenience, we will refer to nodes with the attribute value $x = 1$ as active, and those with $x = 0$ as inactive. The probability that a random node is active is $P(x = 1) = \sum_k P(x = 1|k)p(k)$. The probability that a random neighbor is active is $Q(x = 1) = \sum_k P(x = 1|k)q(k)$. Using Bayes' rule, this can be rewritten as

$$\begin{aligned} Q(x=1) &= \sum_k \frac{P(x=1,k)}{p(k)}\frac{kp(k)}{\langle k\rangle} = \sum_k \frac{P(x=1,k)}{P(x=1)}\frac{kP(x=1)}{\langle k\rangle} \\ &= \frac{P(x=1)}{\langle k\rangle}\sum_k kP(k|x=1) = P(x=1)\frac{\langle k\rangle_{x=1}}{\langle k\rangle}, \end{aligned}$$

where $\langle k\rangle_{x=1}$ is the average degree of active nodes. This quantity and the average degree $\langle k\rangle$ are related via the correlation coefficient $\rho_{kx} = \frac{P(x=1)}{\sigma_x\sigma_k}\left[\langle k\rangle_{x=1} - \langle k\rangle\right]$ (Eq. ??). Hence, the strength of the generalized friendship paradox is

$$Q(x=1) - P(x=1) = \rho_{kx}\frac{\sigma_x\sigma_k}{\langle k\rangle},$$

which is positive when node degree and attribute are positively correlated ($\rho_{kx} > 0$) and increases with this correlation [2].

References

1. Feld SL. Why Your Friends Have More Friends Than You Do. American Journal of Sociology. 1991 May;96(6):1464–1477.

2. Eom YH, Jo HH. Generalized friendship paradox in complex networks: The case of scientific collaboration. Scientific Reports. 2014 Apr;4.
